# Severe Cardiopulmonary Complications and Stroke in a Patient With HIV and Chronic Substance Abuse: A Case Report

**DOI:** 10.7759/cureus.75070

**Published:** 2024-12-03

**Authors:** Waslah M Mansha, Pejmahn Eftekharzadeh, Shahzad Ahmed

**Affiliations:** 1 Cardiothoracic Surgery, Rigshospitalet, Copenhagen, DNK; 2 Cardiology, Lower Bucks Hospital, Bristol, USA

**Keywords:** biventricular failure, eisenmenger, hiv infection, pulmonary arterial hypertension (pah), stroke, substance abuse

## Abstract

This case report presents a 37-year-old male with a complex medical history, including HIV, chronic methamphetamine and cocaine use, and an atrial septal defect, who developed severe pulmonary arterial hypertension (PAH), biventricular failure, and recurrent stroke. The patient was admitted with acute neurological deficits and respiratory failure, which rapidly progressed despite intensive management. Laboratory and imaging studies revealed severe cardiac dysfunction and elevated pulmonary vascular resistance. Despite interventions such as venoarterial extracorporeal membrane oxygenation (VA-ECMO) and intra-aortic balloon support, the patient’s condition deteriorated, necessitating a shift toward palliative care. This case highlights the multifactorial etiology of PAH in the context of polysubstance use and HIV, and underscores the challenges of managing complex cardiovascular and neurological sequelae in patients with multiple comorbidities. Multidisciplinary approaches are essential in such cases to optimize patient outcomes and guide future management strategies.

## Introduction

Pulmonary arterial hypertension (PAH) is a severe and progressive disease characterized by elevated pressures in the pulmonary arteries, which can lead to right ventricular (RV) failure. It can be idiopathic, or it may arise secondary to various conditions such as connective tissue diseases, congenital heart defects, HIV infection, methamphetamine use, and more [1].

Methamphetamine use has increasingly been associated with a variety of cardiovascular complications, including PAH. Chronic methamphetamine use causes persistent sympathetic stimulation, systemic vasoconstriction, and endothelial dysfunction, all of which contribute to elevated pulmonary artery pressures and right ventricular hypertrophy. Methamphetamine may also lead to vascular inflammation, oxidative stress, and injury to vascular smooth muscle cells, further exacerbating pulmonary hypertension [2,3]. Similarly, infection with HIV is known to contribute to pulmonary vascular dysfunction, likely due to chronic inflammation and immune dysregulation that promote vascular remodeling. Studies have indicated that as many as 35% of HIV-positive individuals develop pulmonary hypertension, reflecting the complexity of this pathology [1]. Congenital heart defects, such as atrial septal defect (ASD) and ventricular septal defect (VSD), can also contribute to the development of PAH, especially if left untreated. In these conditions, long-standing left-to-right shunting results in increased blood flow to the pulmonary arteries, which can eventually lead to severe pulmonary arterial hypertension. Methamphetamine use can transiently cause an increase in PAH causing an enlargement of the ASD leading to a right-to-left shunt.

In this report, we present the case of a patient with multifactorial PAH, resulting from chronic methamphetamine and cocaine use, HIV infection, and congenital heart disease. The presence of an ASD further complicated the case, likely exacerbating pulmonary hypertension through a left-to-right shunt. Additionally, the severe impairment of left ventricular ejection fraction (LVEF) underscores the interdependence between left-sided heart failure and secondary pulmonary hypertension, which complicates PAH in patients with multiple risk factors.

## Case presentation

**Figure 1:**
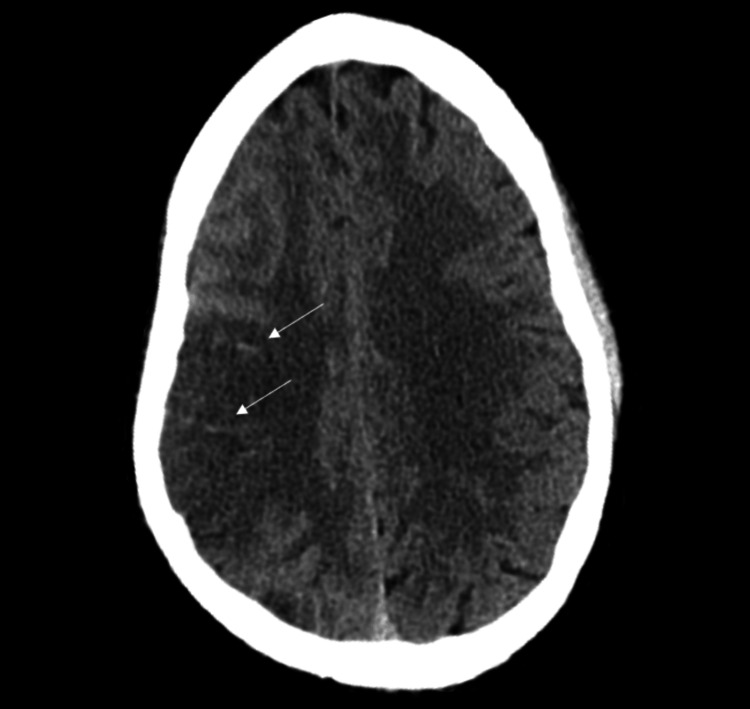
The CT of the head demonstrates right cerebral acute infarcts with gyral hyperdensities. The white arrows point to the areas of hemorrhagic transformation.

**Figure 2:**
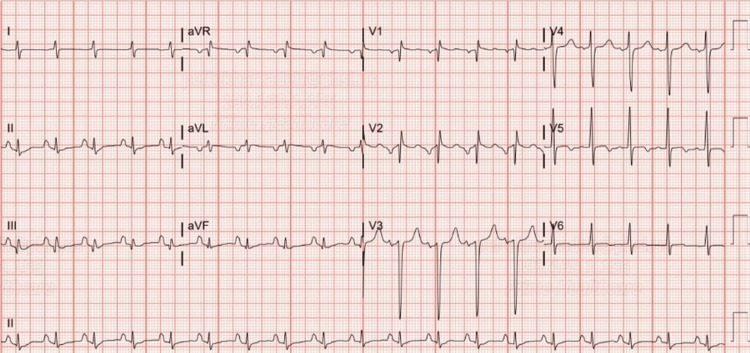
The ECG of the patient reveals an S1Q3T3 pattern.

A 37-year-old male with a medical history of HIV on nucleoside reverse transcriptase inhibitors, polysubstance abuse by snorting cocaine and smoking methamphetamine, alcohol abuse, and depression presented to the ER with the sudden onset of left-sided weakness and facial droop. He had been visiting a mutual friend who had recently passed away. Feeling emotionally distraught, he began consuming large volumes of alcohol and proceeded to use cocaine and methamphetamine. The next day, the patient was found unconscious in his apartment and had noticeable weakness on his left side. Upon presentation to the ER, the patient was found to be in acute hypoxic respiratory failure after his vitals were 133/89 mmHg, 119 beats per minute, 89% oxygen saturation, and 32 breaths per minute and subsequently placed on supplemental oxygen. On physical exam, he was ill-appearing, in distress, tachypneic, and tachycardic. He had clubbing at his fingertips along with flaccid paralysis of his left upper and lower extremities. A stroke code was activated, and a CT head scan revealed right cerebral acute infarcts with gyral hyperdensities, suggesting possible hemorrhagic transformation as shown in Figure 1. Next, an electrocardiogram (ECG) (Figure 2) was notable for an S1Q3T3 pattern demonstrating right ventricular strain, along with prominent P waves (P-pulmonale) demonstrating right atrial enlargement.

**Figure 3:**
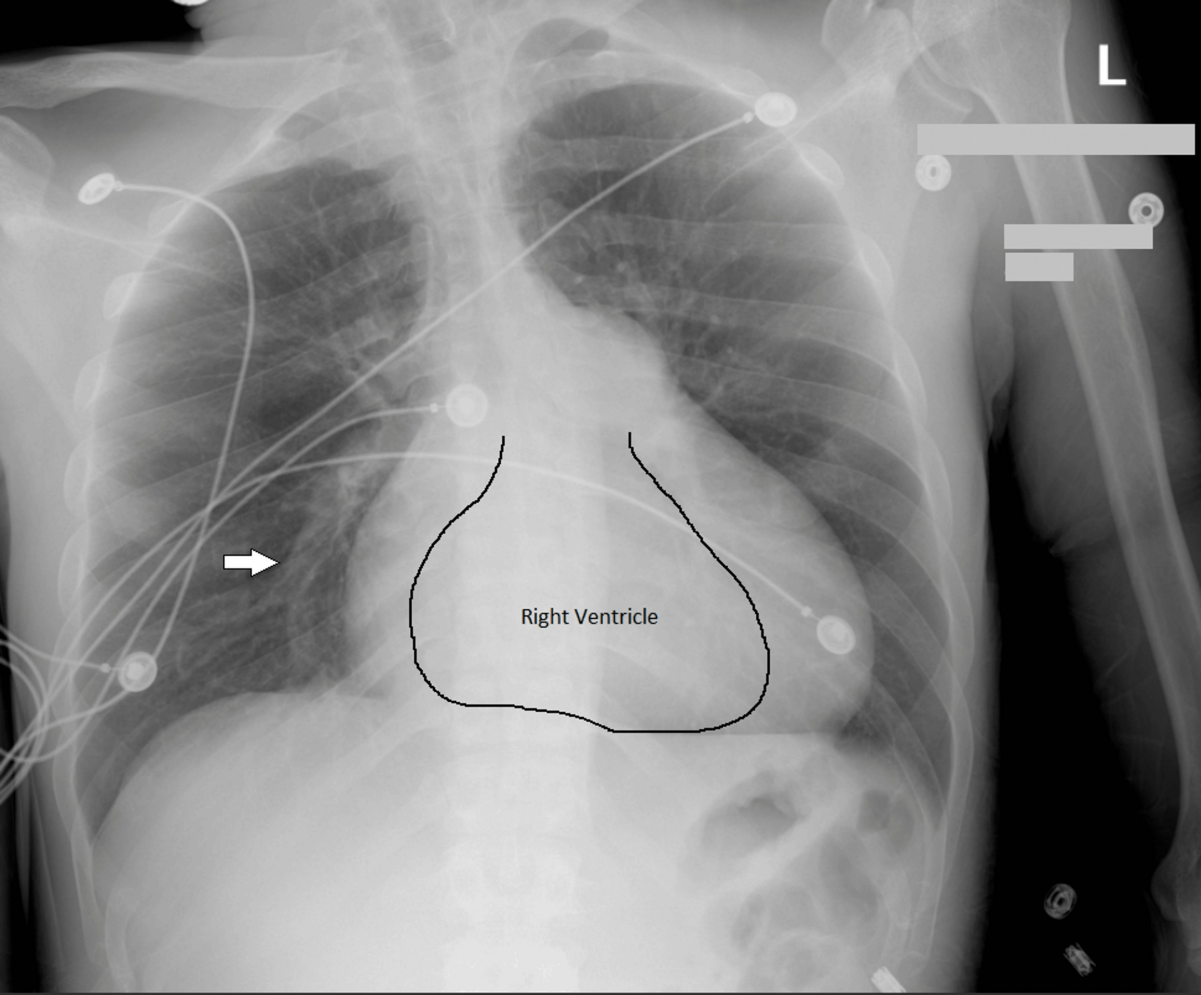
The chest X-ray shows abnormal enlargement of the main pulmonary artery. The white arrow points at the main pulmonary artery.

The patient’s laboratory findings (Table 1) revealed several abnormalities indicative of cardiac stress (troponin increased), renal impairment, and electrolyte imbalance, as shown in the table below. These critical values highlight the patient’s underlying organ dysfunction. Toxicology (Table 2) showed positive for different substances. A chest X-ray showed abnormal enlargement of the main pulmonary artery, consistent with pulmonary hypertension (Figure 3). 

**Table 1:** Laboratory findings showing cardiac injury markers, electrolyte imbalance, and renal function abnormalities

Test	Result	Normal range	Interpretation
Troponin	1.850 ng/L - 2.1061 ng/L	< 14 ng/L	Elevated
Pro-BNP	4099 pg/mL	< 125 pg/ml	Elevated
Sodium (Na)	126 mEq/L	135-145 mEq/L	Hyponatremia
Blood urea nitrogen (BUN)	22 mg/dL	7-20 mg/dL	Elevated
Creatinine	1.26 mg/dL	0.6-1.2 mg/dL	Slight acute elevated
White blood cells (WBC)	10.3 x 10^9^/L	4.0-11.0 x 10^9^/L	Normal
Red blood cells (RBC)	6.11 x 10^12^/L	4.5-5.9 x 10^12^/L	Elevated
Hemoglobin (Hb)	19.1 g/dL	13.5-17.5 g/dL	Elevated likely from chronic hypoxia and secondary erythrocytosis
Hematocrit (Hct)	56.5 %	38.3% - 48.6 %	Elevated likely from chronic hypoxia and secondary erythrocytosis
Mean corpuscular volume (MCV)	92.5 fL	80-100 fL	Normal
Red cell distribution width (RDW)	15%	11.5% - 14.5%	Elevated
Platelets	223 x 10^9^/L	150-450 x 10^9^/L	Normal
Neutrophils	75%	40% - 70%	Elevated
Lymphocytes	20%	20% - 40%	Normal
Monocytes	4.3%	2% - 8%	Normal
Eosinophils	0 %	1% - 4%	Low
Basophils	0.4 %	< 1%	Normal

**Table 2:** Toxicology report

Substance	Result
Amphetamine	Positive
Cocaine	Positive
Amphetamine	Positive
Barbiturate	Negative
Benzodiazepine	Negative
Cannabinoid	Negative
Cocaine	Negative
Opiate	Negative
Phencyclidine (PCP)	Negative
Fentanyl screen	Negative

**Figure 4:**
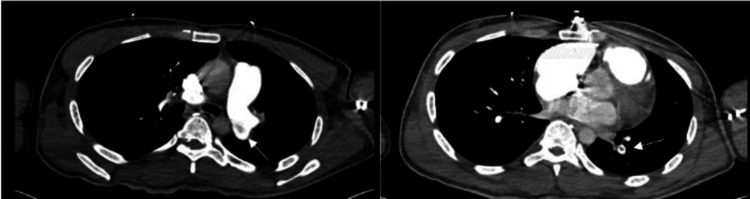
The CTA of the chest reveals pulmonary filling defect within the left main pulmonary artery. The white arrow points at the pulmonary embolism. CTA: Computed tomography angiogram

Computed tomography angiogram (CTA) of the chest (Figure 4) indicated a pulmonary filling defect within the left main pulmonary artery extending into lobar and segmental branches, consistent with pulmonary embolism (Figure 4). Additionally, atelectasis with consolidative opacities was observed in the left lower lobe, alongside focal bullae/blebs in the right middle lobe. 

**Figure 5:**
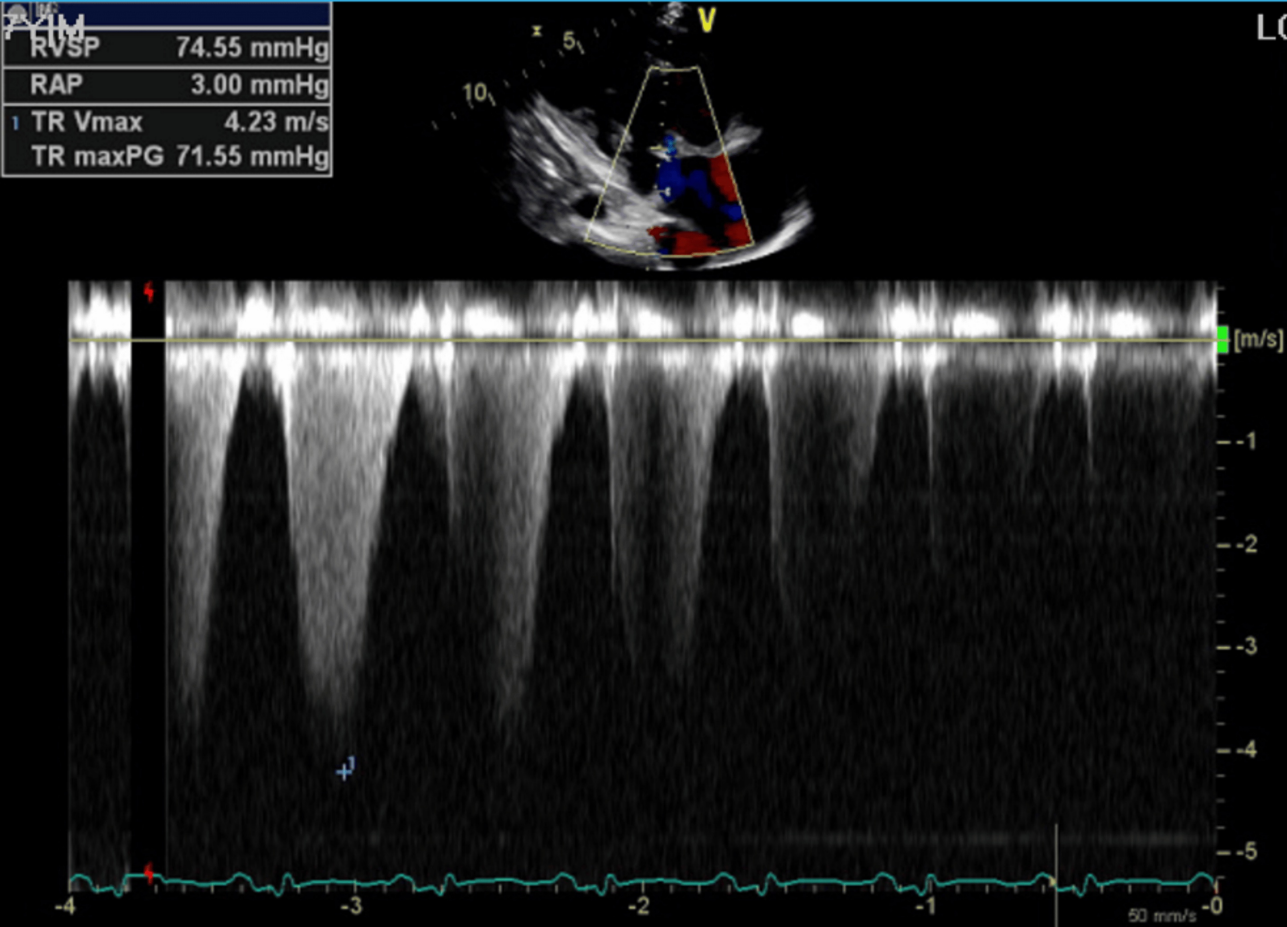
Echocardiogram showing elevated right ventricular systolic pressure (RVSP), indicative of pulmonary hypertension.

**Figure 6:**
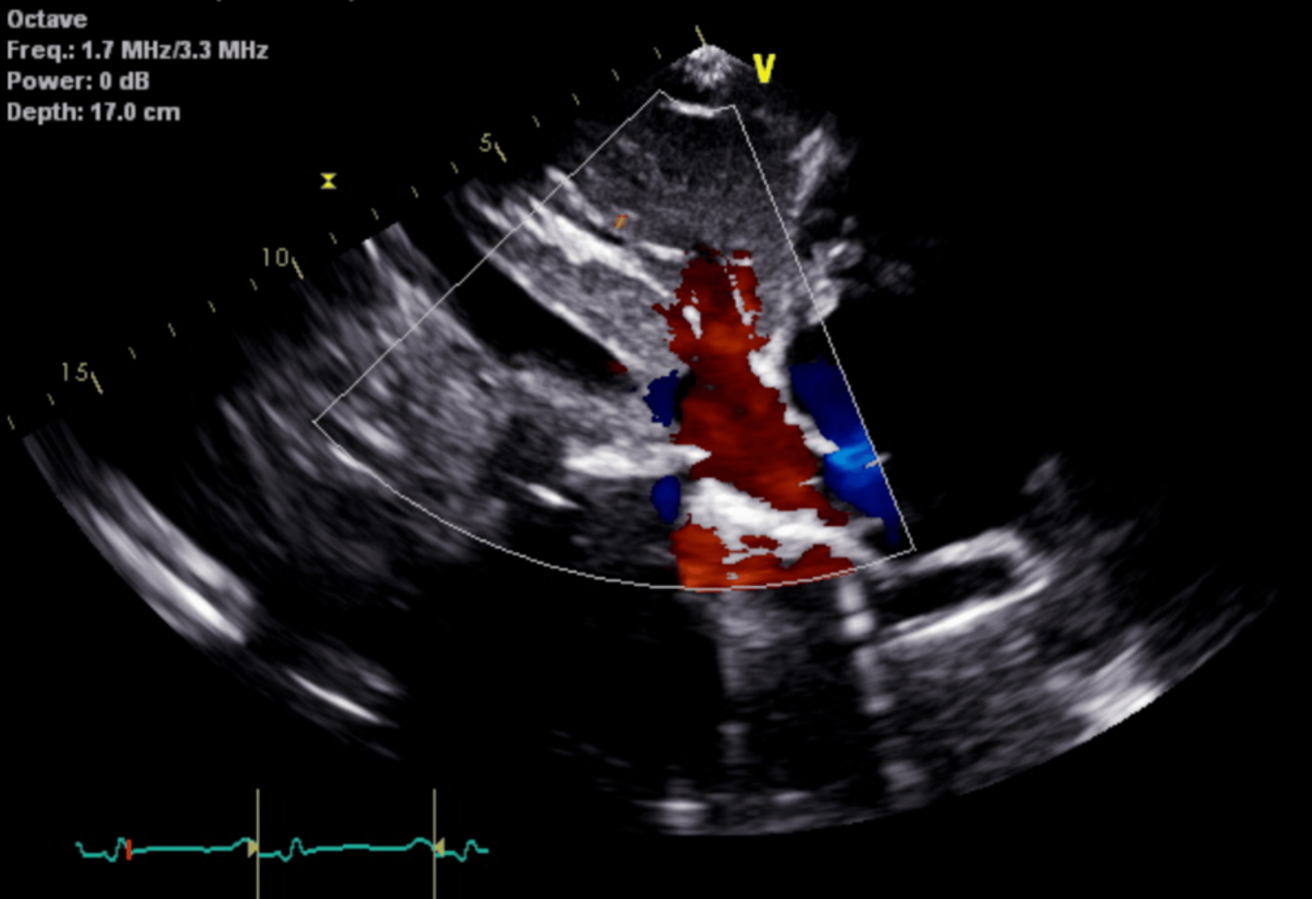
Echocardiogram demonstrates hepatic vein flow reversal, a hallmark of severe tricuspid regurgitation and significant right ventricular overload.

**Figure 7:**
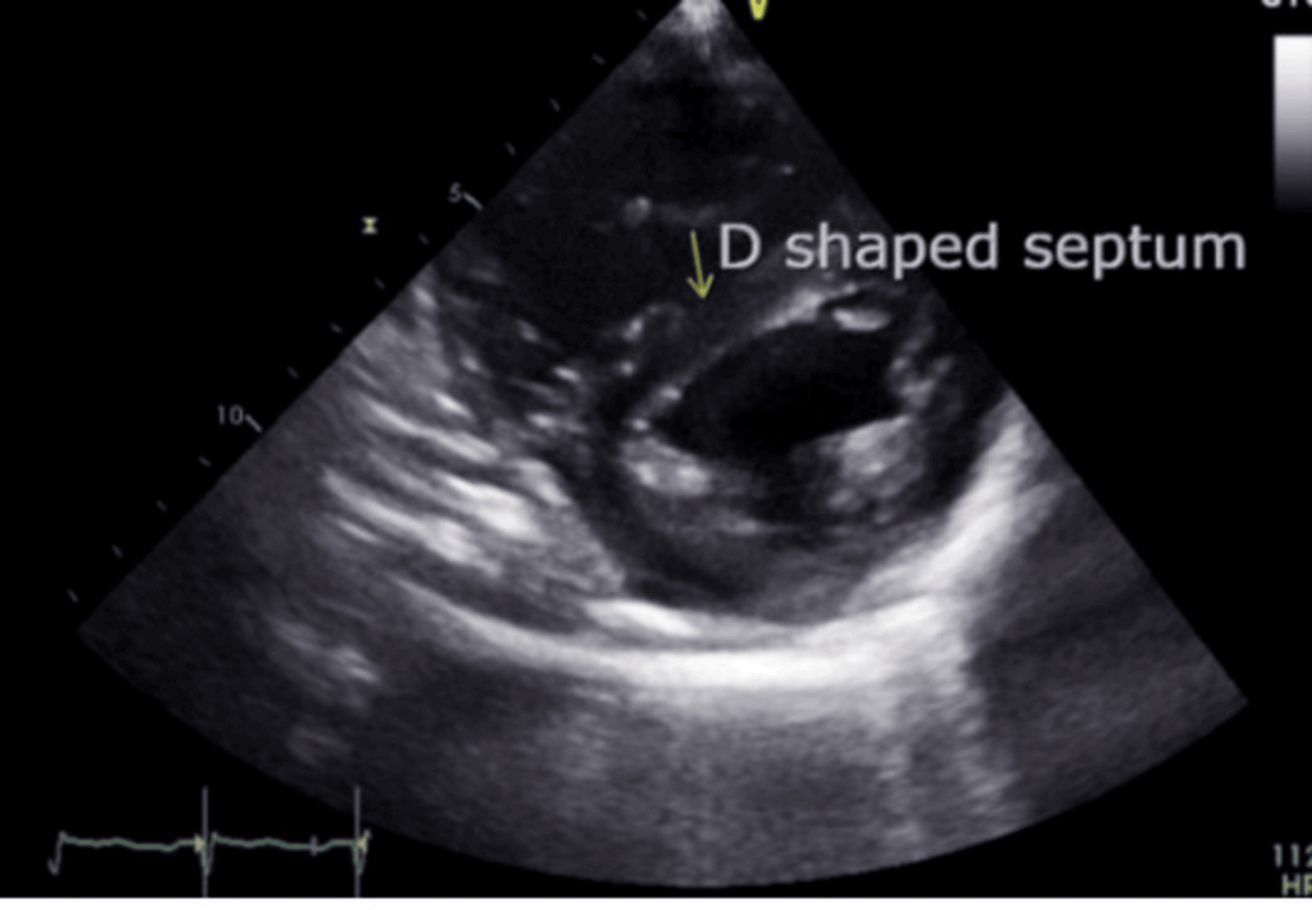
Echocardiogram shows the D-configuration, characteristic of right ventricular overload in the context of elevated pulmonary arterial pressure.

**Figure 8:**
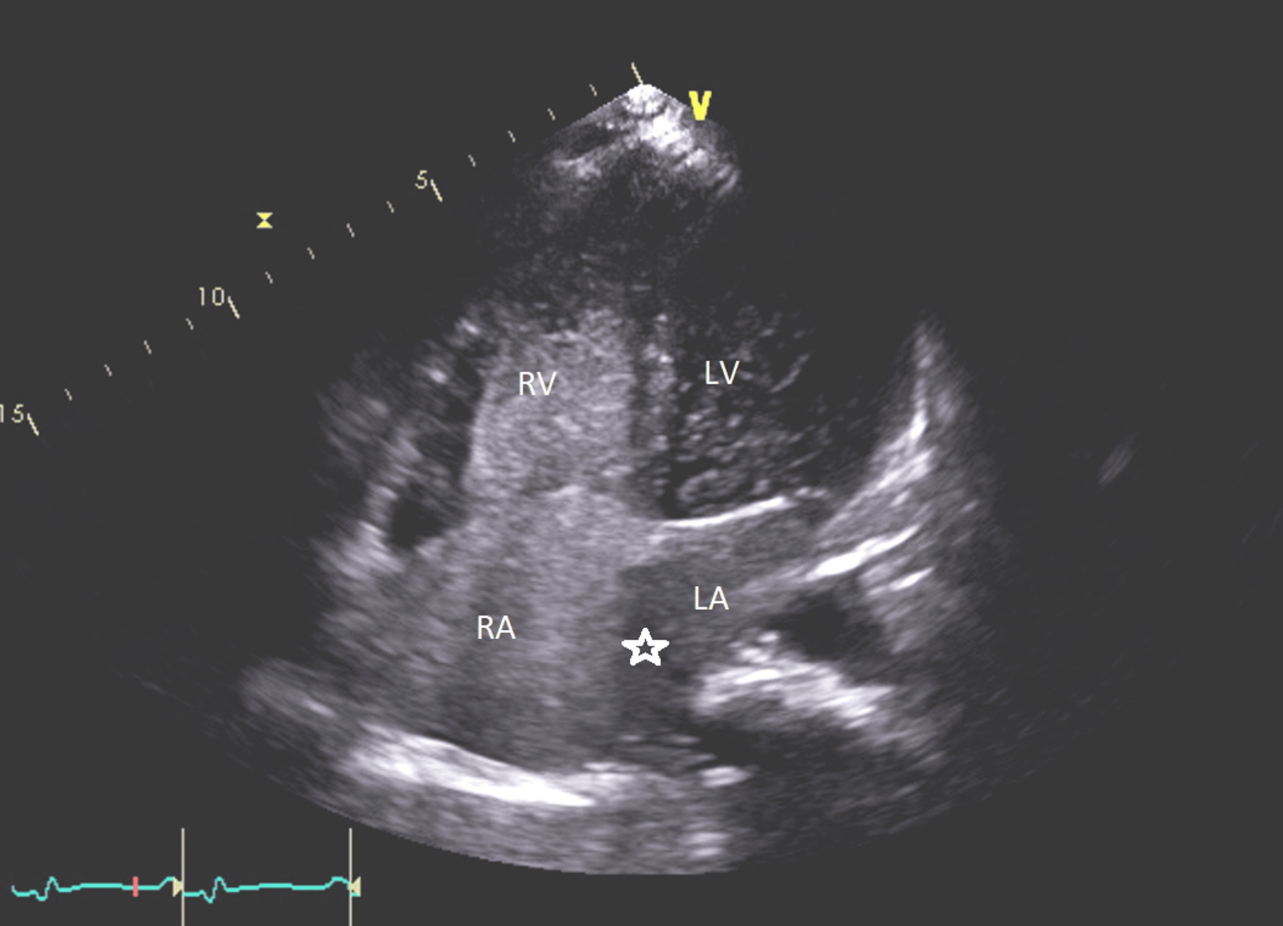
The apical four-chamber view on echocardiogram. Notice how the bubbles deviate from right atrium to left atrium indicating a positive bubble study. This demonstrates an interatrial septal defect. RA: Right atrium, RV: Right ventricle, LA: Left atrium, LV: Left ventricle, Star: Interatrial septal defect

Echocardiography demonstrated a severely reduced left ventricular ejection fraction (LVEF) of 25% to 30% with smoke in the left ventricle, global hypokinesis, severe right atrial dilation, mild right ventricular dilation and hypertrophy, mildly reduced right ventricular systolic function, moderate to severe tricuspid regurgitation (Figure 5), and an aneurysmal atrial septum, with a positive bubble study indicating a right-to-left shunt. The study also revealed hepatic vein flow reversal (Figure 6), characteristic of severe tricuspid regurgitation and indicative of significant right ventricular overload (Figure 6). A "D" shaped septum was consistent with elevated right ventricular pressure (Figure 7). Lastly, the echocardiogram revealed a positive bubble study indicating a right-to-left interatrial shunt (Figure 8).

Right heart catheterization was done to confirm chamber pressures while on a high-flow nasal cannula at 100% fraction of inspired oxygen (FiO2) and 40 L. The right atrial pressure was 9 mmHg, the right ventricular systolic pressure (RVSP) was 75 mmHg, and the mean pulmonary artery pressure was 50 mmHg. The pulmonary capillary wedge pressure was measured at 10 mmHg, and left ventricular end-diastolic pressure was 14 mmHg, resulting in a transpulmonary gradient of 39 mmHg and pulmonary vascular resistance of 11 Woods units. Additional findings included a hemoglobin level of 6.8 g/dL, pulmonary artery saturation of 61.5%, aortic saturation of 98%, a cardiac output of 2.7 L/min, and a cardiac index of 1.52 L/min/m².

A repeat echocardiogram confirmed the findings: an LVEF of 25%, persistent global hypokinesis of the left ventricle, severe right atrial dilation, and an aneurysmal interatrial septum with known patent foramen ovale (PFO)/ASD from the prior bubble study. The echocardiogram also highlighted a low-flow state with smoke observed in the left ventricle.

Clinical course and management

A left cardiac catheterization was done, revealing nonobstructive coronary artery disease with luminal irregularities but no acute occlusions. Over the course of treatment, the echocardiograms indicated a further decline in left ventricular function, with the LVEF dropping to 20% to 25%. The patient was eventually transferred to a tertiary care center for initiation of pulmonary vasodilator therapy after exhausting all resources at the community hospital where he was initially being treated.

At the transferring facility, the patient’s condition worsened, requiring intubation for acute hypoxic respiratory failure. He was placed on vasopressors and subsequently was initiated on an intra-aortic balloon pump for left ventricular failure. After continued failure, the patient was subsequently cannulated on veno-arterial extracorporeal membrane oxygenation (VA-ECMO). 

A repeat transthoracic echocardiogram showed a further decline in LVEF to 10% to 15%. By day 16, the patient underwent an endomyocardial biopsy to further investigate the cause of deterioration of severe biventricular failure, but unfortunately, the procedure was complicated by cardiac tamponade, necessitating emergency sternotomy, and right ventricular repair, which was successful. Epoprostenol was eventually started for PAH but was discontinued as the patient's shock state worsened and the requirement for additional vasopressor therapy precluded the continuation of pulmonary vasodilator therapy.

Outcome

The patient’s right ventricular function slowly began improving, allowing for the decannulation of ECMO and removal of the intra-aortic balloon pump. The patient's clinical status began improving after ECMO and the intra-aortic balloon pump. Unfortunately, the PAH predisposed the patient to a second major ischemic stroke, leading to further deterioration in his neurological status.

## Discussion

Pulmonary hypertension is classified into five subgroups based on etiology. Our patient had numerous comorbidities per multiple WHO classifications of pulmonary hypertension [4]​. Our patient's presentation was consistent with acute on chronic exacerbation of pulmonary hypertension, leading to his acute hypoxic respiratory failure. For example, he had a component of group 1 PAH with a history of HIV, chronic methamphetamine use, and congenital ASD. He had acute nonischemic cardiomyopathy, part of group 2 PAH, which was secondary to illicit drug use with cocaine and methamphetamine. He also may have had evidence of group 4 PAH in the setting of pulmonary embolism. While all the above can cause chronic PAH, his substance abuse may have contributed to the worsening of his PAH, leading to the enlargement of his PFO, thereby further exacerbating his biventricular dysfunction.

After being transferred to the tertiary care center, he had numerous modalities of therapy that targeted each factor of his respiratory compromise. The intra-aortic balloon pump was used to offload left ventricle (LV) strain that developed from his nonischemic cardiomyopathy. He was intubated for acute hypoxic respiratory failure. He was placed on ECMO for acute hypoxic respiratory failure and biventricular failure. Lastly, because his ASD flow was right-to-left, he was not a surgical candidate for fixation of his ASD, as closing will further exacerbate right heart pressures. Other suggested therapies were pulmonary artery vasodilators, such as sildenafil, a phosphodiesterase-5 inhibitor, and epoprostenol, a prostaglandin. However, after its initiation, due to the patient's severe cardiogenic shock status, the medication was discontinued. The patient's clinical status greatly improved after ECMO treatment, and he was The patient's clinical status greatly improved after ECMO treatment, and he was eventually decannulated from it.

The patient has a preexisting congenital defect, the ASD. Throughout his life, the defect was likely small enough that it did not contribute to significant symptomology. However, in this acute setting where methamphetamine can cause or exacerbate PAH, this predisposed him to elevated right-sided heart pressures. The elevated heart pressures, in turn, contributed to an enlargement of his PFO, contributing to his right-to-left shunt. This cascade of events increased blood flow into the left side of the heart, placing more strain on his LV. All this, compounded with his nonischemic cardiomyopathy from cocaine, led to the development of biventricular dysfunction.

The etiology of the stroke is multifactorial. Both methamphetamine and cocaine are risk factors for stroke [5,6]. Methamphetamine causes PAH which predisposes patients to an acute stroke, and cocaine has sympathomimetic effects on the cardiovascular system, causing nonischemic cardiomyopathy [7]. This causes left ventricular dysfunction with a low output state, as evidenced by smoke in the LV on this patient's echocardiogram, leading to potential thrombus formation in the LV that can embolize into the neurovasculature. Though also plausible, any thrombi in the vascular system up to the right atrium could have crossed the ASD to enter the neurovascular system, especially if the PAH was significant enough to enlarge the pre-existing ASD. 

The patient's HIV status likely compounded these effects, increasing the risk for pulmonary vascular complications. While direct infection of pulmonary vascular cells by HIV has not been conclusively established, chronic inflammation and immune dysregulation associated with HIV can lead to vascular remodeling, potentially resulting in PAH. This aligns with epidemiological studies highlighting the role of chronic inflammation as a driver of vascular changes in HIV patients [1].

In patients with similar profiles, a multidisciplinary approach involving cardiology, infectious disease, and addiction specialists is essential for optimizing outcomes. This case highlights the importance of considering all underlying factors contributing to pulmonary hypertension, particularly in patients with complex medical histories. Future research should focus on understanding the mechanisms linking substance use and pulmonary vascular complications, as well as developing targeted therapies for this unique population. 

## Conclusions

This case illustrates the significant challenges associated with managing PAH in patients with concurrent substance use and HIV infection. Chronic congenital defects can be exacerbated by substance use, such as methamphetamine, that can transiently enlarge a patient's congenital ASD as a result of worsening PAH. The interplay of methamphetamine use, pulmonary hypertension, and left heart dysfunction creates a multifaceted clinical scenario that requires a comprehensive, multidisciplinary approach. Understanding the complexities of such cases is critical for improving patient outcomes and guiding future research into the mechanisms linking substance use and pulmonary vascular complications.
